# A process-based model simulating the life-cycle of *Culex pipiens* s.s./*Cx. torrentium* (Diptera: Culicidae) in Germany

**DOI:** 10.1186/s13071-026-07410-4

**Published:** 2026-05-11

**Authors:** Leif Rauhöft, Pride Duve, Sara M. Martins-Afonso, Jöst Hanna, Tatiana Şuleşco, Felix Gregor Sauer, Renke Lühken

**Affiliations:** Bernhard Nocht Institute for Tropical Medicine, Bernhard-Nocht-Straße 74, 20359 Hamburg, Hamburg Germany

## Abstract

**Background:**

Mosquitoes are well known for their ability to transmit pathogens, including various arthropod-borne viruses (arboviruses) of veterinary and medical interest. In Europe, the increasing public health relevance of mosquito-borne pathogens highlights the need to understand how environmental drivers shape mosquito population dynamics relevant to transmission risk. *Culex pipiens* sensu stricto (s.s.) and *Culex torrentium* (collectively referred to here as *Cx. pipiens* s.s./*Cx. torrentium*) are the primary vectors of Usutu virus and West Nile virus in Europe and are commonly found in and around human settlements. The prediction of their spatial-temporal abundance supports early assessment of arbovirus transmission risk and the planning of effective intervention methods, such as vector control.

**Methods:**

A process-based model was developed to simulate the spatial-temporal occurrence of *Cx. pipiens* s.s./*Cx. torrentium* in Germany, with a particular focus on depicting realistic overwintering behaviour, including, diapause induced through photoperiod and temperature in the larval stage. The model output is driven by local temperature and rainfall data provided by the European re-analysis and observations for monitoring data set and evaluated with field data from 106 sampling sites in Germany.

**Results:**

A significant association between relative simulated and observed mosquito abundance was found for 75% of the sampling sites, using site-specific beta regression models. An overall beta mixed-effects model across all sites was also significant (estimate = 2.17, standard error = 0.062, *Z*-value = 35.03, *p*-value < 0.0001, marginal* R*^2^ = 0.4).

**Conclusions:**

This model offers a robust framework for the depiction of the mosquito population dynamics of *Cx. pipiens* s.s./*Cx. torrentium* under current and future climate scenarios, thereby supporting vector surveillance and control strategies across Europe.

**Graphical abstract:**

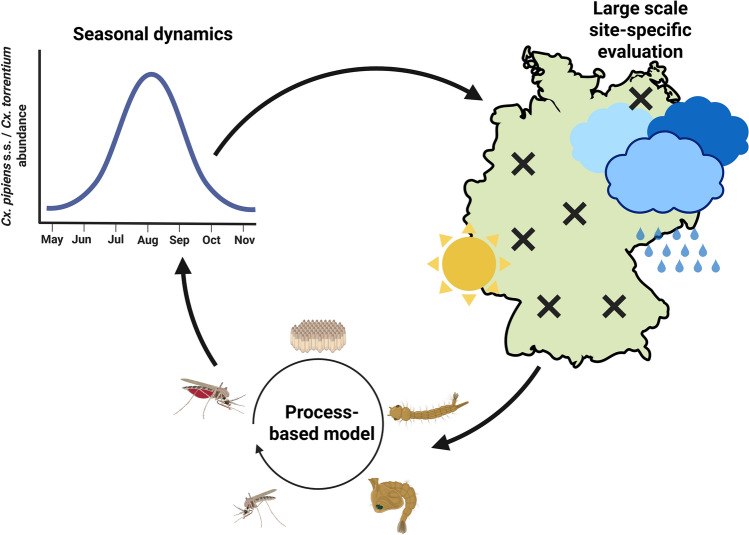

**Supplementary Information:**

The online version contains supplementary material available at 10.1186/s13071-026-07410-4.

## Background

Mosquitoes (Culicidae) are small dipteran insects of global importance due to their role as vectors of numerous pathogens affecting humans and animals. Their life-cycle consists of four distinct stages: egg, larva, pupa and adult. The immature stages develop in aquatic habitats. Female mosquitoes depend on blood meals for egg production, and thus pathogens can be ingested and subsequently transmitted during the next gonotrophic cycle. Male mosquitoes do not feed on blood under natural field conditions. Following a blood meal, female mosquitoes undergo a resting period during which they digest the ingested blood to develop eggs [1], which are then laid in standing water bodies. *Culex pipiens* sensu stricto (*Cx. pipiens* s.s.) and* Culex torrentium* (collectively referred to here as *Cx. pipiens* s.s./*Cx. torrentium*) are very important mosquito vectors of aboviruses of medical and veterinary relevance [2], with a high vector competence for Usutu virus and West Nile virus (WNV) [3, 4]. Since their initial emergence in Germany, where Usutu virus was first detected in 2011 and WNV in 2018, these pathogens have been detected each year in birds, mosquitoes and humans [5–8]. WNV and Usutu virus are mosquito-borne flaviviruses that are maintained in enzootic transmission cycles between mosquitoes and avian hosts, with humans and other mammals acting as incidental dead-end hosts. While most human infections are asymptomatic, both viruses can cause neuroinvasive disease, particularly in elderly and immunocompromised individuals, and they have emerged as significant public health concerns in several European countries. *Culex pipiens* s.s./*Cx. torrentium* are widely distributed and have broad host-feeding patterns, including birds, non-human mammals and humans, making them suitable bridge vectors [9]. These characteristics have resulted in these two mosquito species being a main target for surveillance and risk assessment efforts [10, 11]. However, although *Cx. pipiens* s.s./*Cx. torrentium* are separate species, the females cannot be morphologically differentiated. They are sibling species that frequently occur in sympatry across Europe, with *Cx. pipiens* s.s. being ubiquitous throughout Europe while *Cx. torrentium* abundances increase in more northern and cooler regions. However, each of these species has distinct biological and ecological characteristics. For example, *Cx. pipiens* s.s. is easily captured with CO_2_-baited traps, while *Cx. torrentium* seems to be disproportionately underrepresented in such captures [12]. In addition, *Cx. torrentium* has a higher vector competence for WNV compared to *Cx. pipiens* s.s. [3]. Also, these two species use different overwintering sites, with *Cx. pipiens* s.s. predominant in anthropogenic sites, such as cellars, and *Cx. torrentium* more frequently found in abandoned animal burrows [13].

Entomological monitoring through systematic mosquito collections provides the foundation for identifying the spatial-temporal risk of mosquito-borne pathogen transmission. Field-based mosquito surveillance, including collection using CO_2_-baited traps and subsequent taxonomic identification and arbovirus screening, is logistically demanding and difficult to sustain at high spatial and temporal resolution. Process-based (mechanistic) models describe dynamic systems by representing the biological processes that determine system behaviour rather than relying on statistical correlations. Building on the foundations laid by Lotka [14], these models provide a framework linking environmental drivers to population dynamics. In the study reported here, we used a process-based model to simulate the life-cycle of *Cx. pipiens* s.s./*Cx. torrentium*, using temperature-, precipitation- and photoperiod-dependent equations to capture developmental and mortality rates. This type of model enables the spatial-temporal mosquito abundance to be estimated while at the same time allowing the simulation of changing conditions, such as control measures [15]. Several process-based models for mosquitoes have been published for Europe, with a focus on the Mediterranean region (southern France or Italy [15–18]). These models showed a high level of performance in terms of estimating the temporal mosquito abundance, but they were generally only validated with field data from a few sampling sites or relatively small areas. Consequently, a process-based model for *Cx. pipiens* s.s./*Cx. torrentium*, two of the most important vectors in Central Europe, and a large-scale, site-specific evaluation is missing.

In this work, we developed and validated a process-based model for *Cx. pipiens* s.s./*Cx. torrentium* in Germany. To achieve this, we adjusted the generic model for *Aedes albopictus* originally proposed by Cailly et al. [15] and later extended to further species, including “*Culex pipiens*”, by Ezanno et al. [16]. We extend the model by replacing predefined seasonal start and end dates with explicit, photoperiod- and temperature-driven diapause dynamics. The model further incorporates sex-differentiated life stages and revised temperature-dependent transition and mortality functions, allowing realistic representation of overwintering behaviour under temperate European conditions.

The aim of this study was to develop and validate, for the first time in Germany, a process-based model for *Cx. pipiens* s.s./*Cx. torrentium*, perform site-specific validation to capture local population dynamics, compare the performance of this process-based model to that of a temperature-dependent correlative model and assess the relative importance of model parameters on overall mosquito dynamics using a sensitivity analysis.

## Methods

### Mathematical model for the life cycle of *Cx. pipiens* s.s./*Cx. torrentium*

**Fig. 1:**
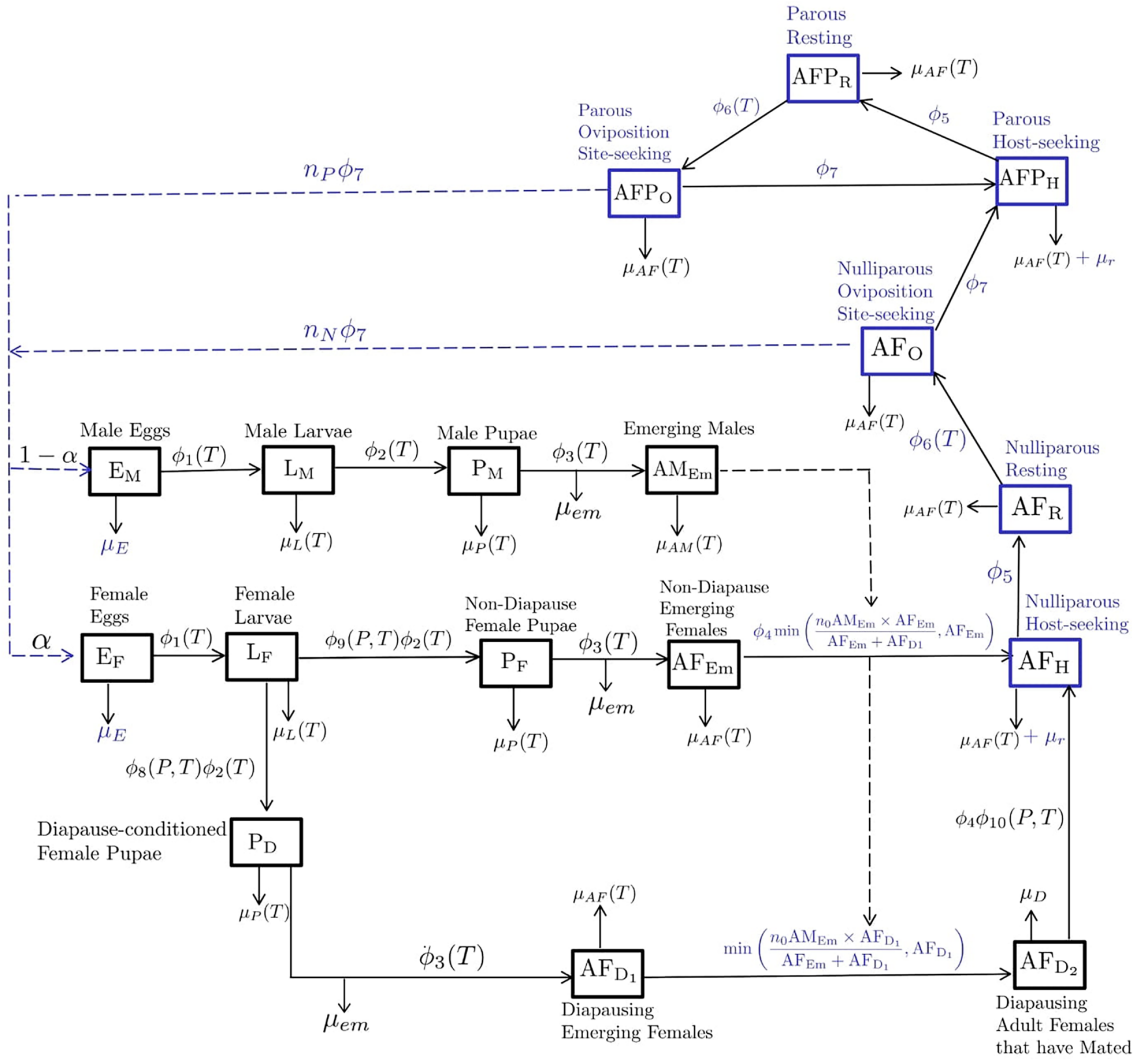
Flow chart of the process-based model for the life-cycle of mosquitoes* Culex pipiens* sensu stricto and* Culex torrentium* (*Cx. pipiens* s.s./*Cx. torrentium*). Boxes represent compartments or different life stages. Solid lines represent the development from one stage to another and dashed lines represent the interactions between the different compartments. Previously published compartments, transition and mortality rates are highlighted in blue. For specific definitions, see Table 1 and section Mathematical model for the life cycle of *Cx. pipiens* s.s./*Cx. torrentium*

The model consists of different compartments (Fig. 1) for mosquito abundance in aquatic stages. These are male and female eggs ($E_M$, $E_F$); larvae ($L_M$, $L_F$); pupae ($P_M$, $P_F$) and diapause-conditioned pupae ($P_D$); and adult stages: emerging males and females ($AM_{Em}$; $AF_{Em}$), nulliparous host-seeking females ($AF_H$); nulliparous resting females ($AF_R$) and nulliparous oviposition site-seeking females ($AF_O$); parous host-seeking females ($AFP_H$); parous resting females ($AFP_R$) and parous oviposition site-seeking females ($AFP_O$); and diapausing stages: emerging adult females ($AF_{D1}$) and overwintering adult females ($AF_{D2}$; Eq. 1).

**Fig. 2:**
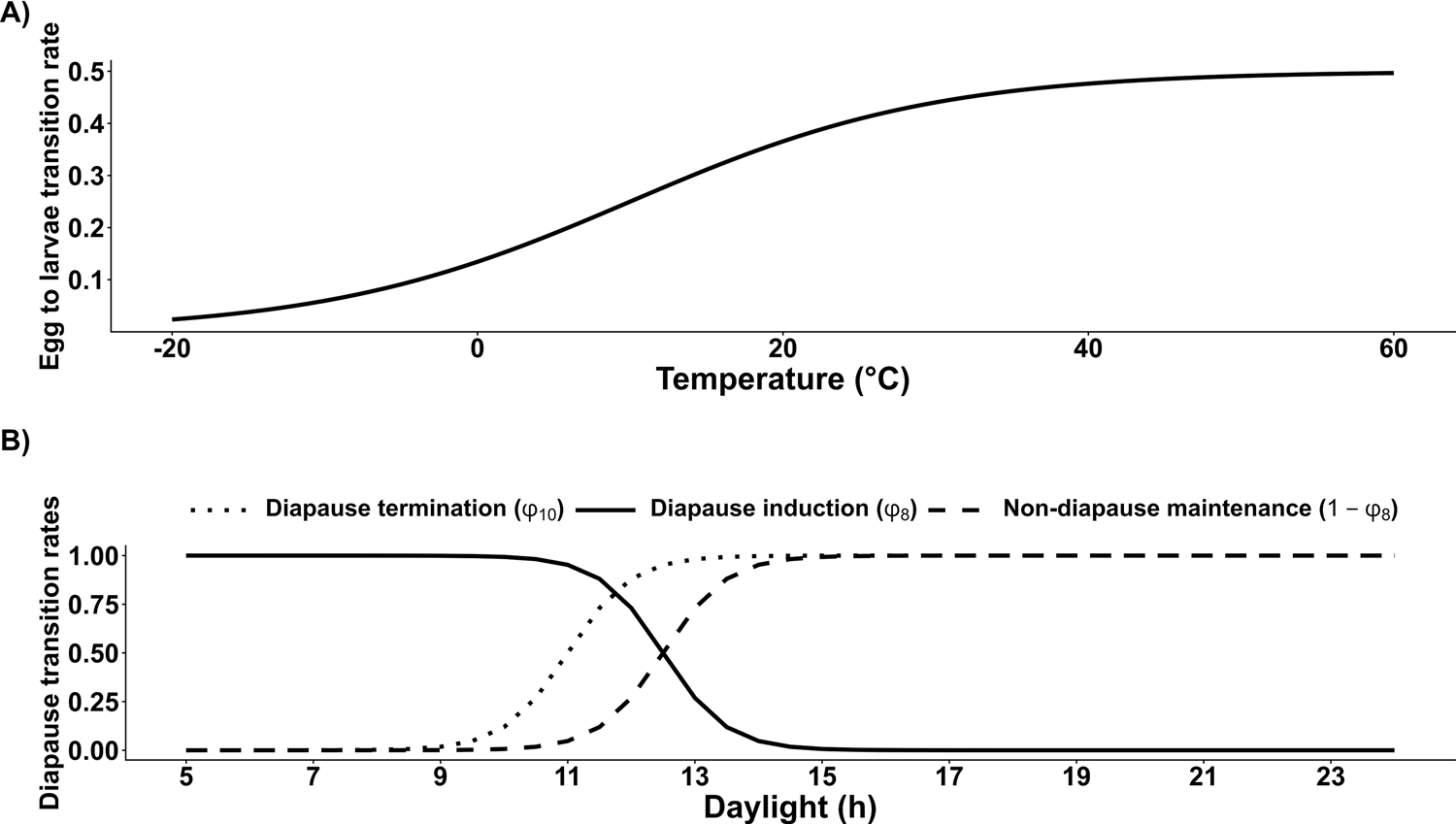
**A** Newly developed temperature-dependent transition rates. **B** Newly developed photoperiod-dependent transition rates

**Fig. 3:**
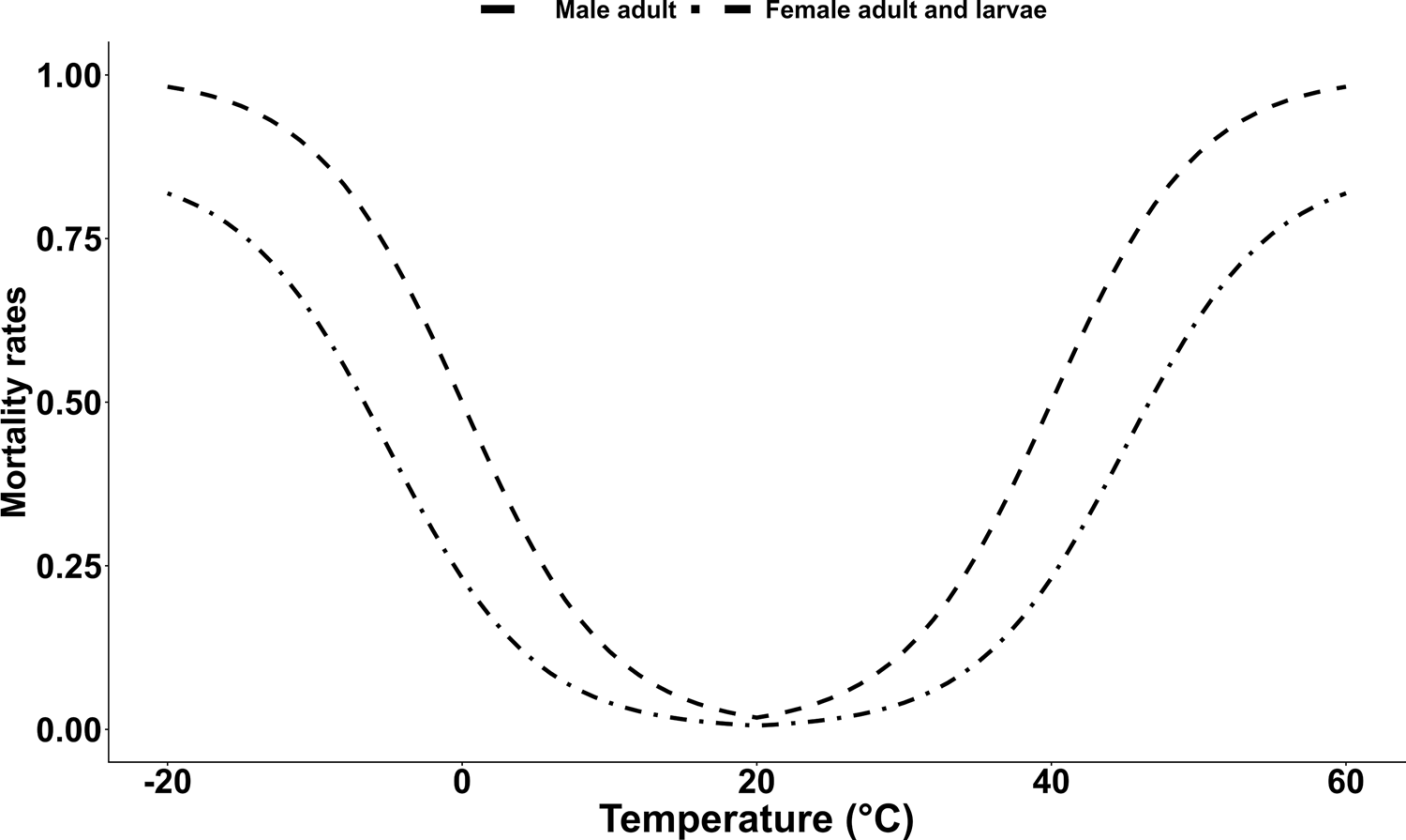
Temperature-dependent mortality rates

 The transition between the aquatic stages is temperature-dependent ($\phi _1(T)$, $\phi _2(T)$, $\phi _3(T)$; Eq. 2; Fig. 2a). Previous laboratory and semi-field studies suggest that diapause is induced at photoperiods < 15 h of light and temperatures lower than 20 °C [19, 20]. This is represented in our temperature- and photoperiod-dependent function $\phi _8(P, T)$ inducing diapause (Fig. 2b; Eq. 3). Adult females that do not enter diapause remain in the reproductive (non-diapausing) state, which is governed by the temperature- and photoperiod-dependent non-diapause maintenance rate $\phi _9(P, T)$. The rate of diapause termination is also temperature- and photoperiod-dependent ($\phi _{10}(P,T)$; Eq. 4; [21]). The development of eggs begins at temperatures exceeding $T_{Ag}$. Development is over when the cumulative daily temperatures exceeds $TDD_{Ag}$. Additionally, temperature-dependent mortality rates were utilised for larvae and pupae ($\mu _L(T)$, $\mu _P(T)$; Eq 5; Fig. 3). Rainfall increases the environmental carrying capacity for larvae ($K_L$) and pupae ($K_P$) by increasing the standard environmental carrying capacities ($\kappa _l$, $\kappa _p$) using the normalised rainfall over a period of 14 days (Eq 6). The competition for resources within the breeding site is taken into account by the density-dependent larval mortality of $\mu _L(T)\left[ 1+\frac{\mu _L(T)}{K_L}\right]$ [16, 22, 23].

The pupal density dependence was previously combined in an exponential decay function using the mortality at emergence ($\mu _{em}$) as scaling factor. We propose that the mortality at emergence ($\mu _{em}$) is not a matter of resources since pupae do not take up any nutrients. Consequently, the mortality at emergence can only be a stochastic event or a matter of actual space restriction by density. We thereby replaced the scaling factor with $\beta$ and strongly reduced it compared to $\mu _{em}$. The mortality at emergence was kept outside of the exponential decay function.

A proportion $\alpha$ of mosquito eggs are female while a proportion of ($1-\alpha$) are male. Based on the assumption that all female mosquitoes mate before seeking a host, emerging females ($AF_{Em}$) and emerging diapausing females ($AF_{D1}$) mate before the females progress either to the host-seeking stage ($AF_H$) or the overwintering stage ($AF_{D2}$). As also implemented by Ezanno et al. [16], it is assumed that females mate only once. In contrast, males can mate several times. Asman [24] showed that under laboratory conditions male *Cx. tarsalis* mosquitoes can inseminate up to eight females over 21 days. Thereby, the number of females which get fertilised by males is highly dependent on the population density of sexually active male mosquitoes [25]. When sexually active males are scarce in the environment, the number of inseminated females that progress to the host-seeking stage is proportional to the number of available males multiplied by the number of females each male can inseminate. This is denoted as $n_0AM_{Em}$, where $n_0$ is the number of females each male inseminates. After mating, female mosquitoes progress to the host-seeking stage at a rate of $\phi _4$. Blood-feeding induces the resting stage at a rate of $\phi _5$ before females start seeking an oviposition site to lay eggs at a rate of $\phi _6(T)$ (Eq 7). Female mosquitoes become parous after depositing eggs at a rate of $\phi _7$. The cycle repeats until the mosquitoes die and are removed from the population. During the diapausing stage, the overwintering females die at a rate of $\mu _D$. Female adult mosquitoes in general die at a rate of $\mu _{AF}(T)$ (Eq 5), while for host-seeking females $AF_H$ and $AFP_H$, an additional host seeking mortality $\mu _r$ is added. Adult males are shown to have shorter longevity especially during the highest and lowest temperatures [26]. Therefore, an additional mortality rate for males $\mu _{AM}(T)$ (Eq. 8) was generated based on the general adult mortality rate but was more constrained in the temperature range (Fig. 3).

1 $$\begin{aligned} \begin{aligned} \frac{dE_F}{dt}&= \alpha \cdot \phi _7 \left[ n_P AFP_O + n_N AF_O \right] - \left[ \phi _1(T) + \mu _E(T)\right] E_F, \\ \frac{dE_M}{dt}&= (1 - \alpha ) \phi _7 \left[ n_P AFP_O + n_N AF_O \right] - \left[ \phi _1(T) + \mu _E(T)\right] E_M, \\ \frac{dL_F}{dt}&= \phi _1(T)\cdot E_F - \left[ \phi _2(T) + \mu _L(T) \left( 1 + \frac{L_F + L_M}{K_L}\right) \right] L_F, \\ \frac{dL_M}{dt}&= \phi _1(T)\cdot E_M - \left[ \phi _2(T) + \mu _L(T) \left( 1 + \frac{L_F + L_M}{K_L}\right) \right] L_M, \\ \frac{dP_F}{dt}&= \phi _2(T) \cdot \phi _9(P,T)\cdot L_F - [\phi _3(T) + \mu _P(T)] P_F, \\ \frac{dP_M}{dt}&= \phi _2(T)\cdot L_M - [\mu _P(T) + \phi _3(T)] P_M, \\ \frac{dP_D}{dt}&= \phi _2(T)\cdot \phi _8(P,T)\cdot L_F - [\mu _P(T) + \phi _3(T)] P_D, \\ \frac{dAM_{Em}}{dt}&= \phi _3(T)\cdot P_M\cdot (1-\mu _{em})\cdot e^{\displaystyle -\beta \left( 1 + \frac{P_M+P_D+P_F}{K_P}\right) } - \mu _{AM}(T)\cdot AM_{Em}, \\ \frac{dAF_{Em}}{dt}&= \phi _3(T)\cdot P_F\cdot (1-\mu _{em})\cdot e^{\displaystyle -\beta \left( 1 + \frac{P_M+P_D+P_F}{K_P}\right) } - \phi _4\min \left\{ n_0 AM_{Em} \frac{AF_{Em}}{AF_{D1}+AF_{Em}}, AF_{Em}\right\} - \mu _{AF}(T)\cdot AF_{Em}, \\ \frac{dAF_{D1}}{dt}&= \phi _3(T)\cdot P_D\cdot (1-\mu _{em})\cdot e^{\displaystyle -\beta \left( 1 + \frac{P_M+P_D+P_F}{K_P}\right) } - \min \left\{ n_0 AM_{Em} \frac{AF_{D1}}{AF_{D1}+AF_{Em}}, AF_{D1}\right\} - \mu _{AF}(T)\cdot AF_{D1}, \\ \frac{dAF_{D2}}{dt}&= \min \left\{ n_0 AM_{Em} \frac{AF_{D1}}{AF_{D1}+AF_{Em}}, AF_{D1}\right\} - [\phi _4\cdot \phi _{10}(P,T)+\mu _{D}]AF_{D2}, \\ \frac{dAF_H}{dt}&= \phi _4\min \left\{ n_0 AM_{Em} \frac{AF_{Em}}{AF_{D1}+AF_{Em}}, AF_{Em}\right\} + \phi _4\cdot \phi _{10}(P,T)\cdot AF_{D2}- [\phi _5+\mu _{r}+\mu _{AF}(T)]AF_H, \\ \frac{dAF_{R}}{dt}&= \phi _5\cdot AF_H - \left[ \phi _6(T) + \mu _{AF}(T)\right] AF_R, \\ \frac{dAF_O}{dt}&= \phi _6(T)\cdot AF_R - \left[ \phi _7 + \mu _{AF}(T)\right] AF_O, \\ \frac{dAFP_H}{dt}&= \phi _7\cdot [AF_O + AFP_O] - \left[ \phi _5 +\mu _{r}+ \mu _{AF}(T)\right] AFP_H, \\ \frac{dAFP_R}{dt}&= \phi _5\cdot AFP_H - \left[ \phi _6(T) + \mu _{AF}(T)\right] AFP_R, \\ \frac{dAFP_O}{dt}&= \phi _6(T)\cdot AFP_R - \left[ \phi _7 + \mu _{AF}(T)\right] AFP_O. \end{aligned} \end{aligned}$$

2 $$\begin{aligned}&\phi _1(T), \phi _2(T), \phi _3(T)&= \frac{0.5}{1+ e^{\displaystyle 0.1(10-T)}} \end{aligned}$$

3 $$\begin{aligned} \phi _8(P,T)&= {\left\{ \begin{array}{ll} \displaystyle \frac{1}{1+ e^{ \displaystyle \frac{12.5-P}{-0.5}}} & \quad \text {if } T<15^\circ \text {C} \\ 0 & \quad \text {otherwise} \end{array}\right. } \end{aligned}$$

4 $$\begin{aligned} \phi _{10}(P,T)&= {\left\{ \begin{array}{ll} \displaystyle \frac{ e^{\displaystyle \frac{P - 11}{0.5}}}{1+ e^{\displaystyle \frac{P - 11}{0.5}}} & \quad \text {if } T>6^\circ \text {C} \\ 0 & \quad \text {otherwise} \end{array}\right. } \end{aligned}$$

5 $$\begin{aligned} \mu _L(T), \mu _P(T),\mu _{AF}(T)&= {\left\{ \begin{array}{ll} \displaystyle \frac{0.86\phantom {\frac{1}{1}}}{1 + e^{\displaystyle 0.2(45-T)}} & \quad \text {if } T > 20^\circ \text {C} \\ \displaystyle \frac{0.86\phantom {\frac{1}{1}}}{1 + e^{\displaystyle \frac{5+T}{5}}} & \quad \text {otherwise} \end{array}\right. } \end{aligned}$$

6 $$\begin{aligned} K_L(R), K_P(R)&= \kappa _X \cdot \left( R_{\textrm{norm}}(R) + 1\right) \quad X \in \{l, p\} \end{aligned}$$

7 $$\begin{aligned} \phi _6(T)&= {\left\{ \begin{array}{ll} \displaystyle \frac{T-T_{Ag}}{TDD_{Ag}} & \quad \text {if } T>T_{Ag} \\ 0 & \quad \text {otherwise} \end{array}\right. } \end{aligned}$$

8 $$\begin{aligned} \mu _{AM}(T)&= {\left\{ \begin{array}{ll} \displaystyle \frac{1\phantom {\frac{1}{1}}}{1+e^{\displaystyle 0.2(40-T)}} & \quad \text {if } T > 20^\circ \text {C} \\ \displaystyle \frac{1\phantom {\frac{1}{1}}}{1+e^{\displaystyle - \frac{T}{5}}} & \quad \text {otherwise} \end{array}\right. } \end{aligned}$$

#### Model initialisation and parameterisation

Following previous implementations of climate-driven mosquito population models, simulations were initialised with 10^6^ male and female eggs each ($E_M$, $E_F$) to avoid numerical extinction at the start of the simulation period [17]. In addition, small non-zero numbers (1000 specimens each) of emerging females ($AF_{Em}$) and emerging diapausing females ($AF_{D1}$) were assigned at initialisation to ensure that all coupled stage transitions in the model equations were defined from the first time step. The remaining compartments initially contain zero specimens, allowing population dynamics to develop from temperature-, rainfall-, and photoperiod-driven processes. Simulations from each sampling site were initialised using site-specific environmental data starting from 1 January 2015, which is 1 year prior to the first mosquito field sampling, to ensure that model outputs at the time of validation were not influenced by the choice of initial conditions. The simulations ended on 31 December 2023.

The majority of model parameters were derived from published literature (Table 1). Existing temperature-dependent development and mortality functions from studies conducted in Mediterranean regions led to unrealistic population collapse under temperate climatic conditions and were therefore unsuitable for Central and Northern Europe.

To address limitations of temperature-dependent mortality and developmental functions derived primarily from Mediterranean studies, biologically plausible functions were reformulated for temperate climatic conditions. The functional shape and range of the temperature–mortality relationship were informed by published experimental studies on *Cx. pipiens* s.s./*Cx. torrentium* (e.g. [27, 28]) and supported by independent survival (Additional file 1: Figure S1) and development (Additional file 1: Figure S2) experiments conducted by the authors under fluctuating temperature regimes in a climate chamber. These sources were used to define biologically realistic response shapes, while the viable temperature range was broadened to reflect survival under Europe-wide climatic conditions and heterogeneous environments.

This approach is consistent with recent assessments highlighting the limited transferability of temperature-dependent parameters derived under restricted climatic conditions and the need for biologically plausible extrapolation when applying mechanistic models across broader environmental gradients [29]. Model parameters were defined a priori to ensure biologically plausible seasonal timing, without reliance on calibration to field-derived abundance data.

**Table 1:** Table of all model parameters, their definitions, values or functional forms, and biological origin

Parameter	Definition	Value	Reference	Origin
$\phi _1(T)$	Egg development rate	Eq. 2	[16]^a^	*Cx. pipiens* s.s.
$\phi _2(T)$	Larval development rate	Eq. 2	[16]^a^	*Cx. pipiens* s.s.
$\phi _3(T)$	Pupal development rate	Eq. 2	[16]^a^	*Cx. pipiens* s.s.
$\phi _4$	Host-seeking rate	0.143	[16]	*Cx. pipiens* s.s./*Cx. torrentium*
$\phi _5$	Resting rate	0.885	[16]	*Cx. pipiens* s.s./*Cx. torrentium*
$\phi _6(T)$	Oviposition site-seeking rate	Eq. 7	[16]	*Cx. pipiens* s.s./*Cx. torrentium*
$\phi _7$	Egg-laying rate	0.2	[16]	*Cx. pipiens* s.s./*Cx. torrentium*
$\phi _8(P,T)$	Diapause induction rate	Eq. 3	[19, 20]	*Cx. pipiens* s.l.
$\phi _9(P,T)$	Non-diapause maintenance rate	$1 - \phi _8(P,T)$	[19, 20]	*Cx. pipiens* s.l.
$\phi _{10}(P,T)$	Diapause termination rate	Eq. 4	[21]	*Cx. pipiens* s.l.
$\mu _E$	Egg mortality rate	0.0262	[16]	*Cx. pipiens* s.s./*Cx. torrentium*
$\mu _L(T)$	Larval mortality rate	Eq. 5	[27, 28]^a^	*Cx. pipiens* s.s.
$\mu _P(T)$	Pupal mortality rate	Eq. 5	[27, 28]^a^	*Cx. pipiens* s.s.
$\mu _{AF}(T)$	Mortality rate of female adult mosquito	Eq. 5	[27, 28]	*Cx. pipiens* s.s.
$\mu _r(T)$	Host-seeking mortality	0.08	[16]	*Cx. pipiens* s.s./*Cx. torrentium*
$\mu _{AM}(T)$	Mortality rate of male adult mosquito	Eq. 8	[27, 28 ]	*Cx. pipiens* s.s.
$\mu _D(T)$	Mortality rate of diapausing female mosquito	0.02	[30]	*Cx. pipiens* s.s.
$\alpha$	Sex ratio at emergence stage	0.5	[16]	*Cx. pipiens* s.s./*Cx. torrentium*
$\beta$	Strength of pupal density dependence	0.003	[a]	*Cx. pipiens* s.s.
$T_E$	Minimal temperature for egg maturation ($^\circ \textrm{C}$)	9.8	[16, 31, 32]	*Cx. pipiens* s.l.
$TDD_{Ag}$	Degree-days for egg maturation ($^\circ \textrm{C}$)	64.4	[16, 31, 32]	*Cx. pipiens* s.l.
$\kappa _l$	Larval standard environmental carrying capacity	8 × 10^8^	[16, 31, 32]	*Cx. pipiens* s.l.
$\kappa _p$	Pupal standard environmental carrying capacity	10^7^	[16, 31, 32]	*Cx. pipiens* s.l.

*Cx. Culex*,* s.l.* sensu lato, * s.s.* sensu stricto

^a^ Functional forms or parameter magnitudes were informed by laboratory experiments conducted under fluctuating temperature regimes and supported by published studies

### Validation

**Fig. 4:**
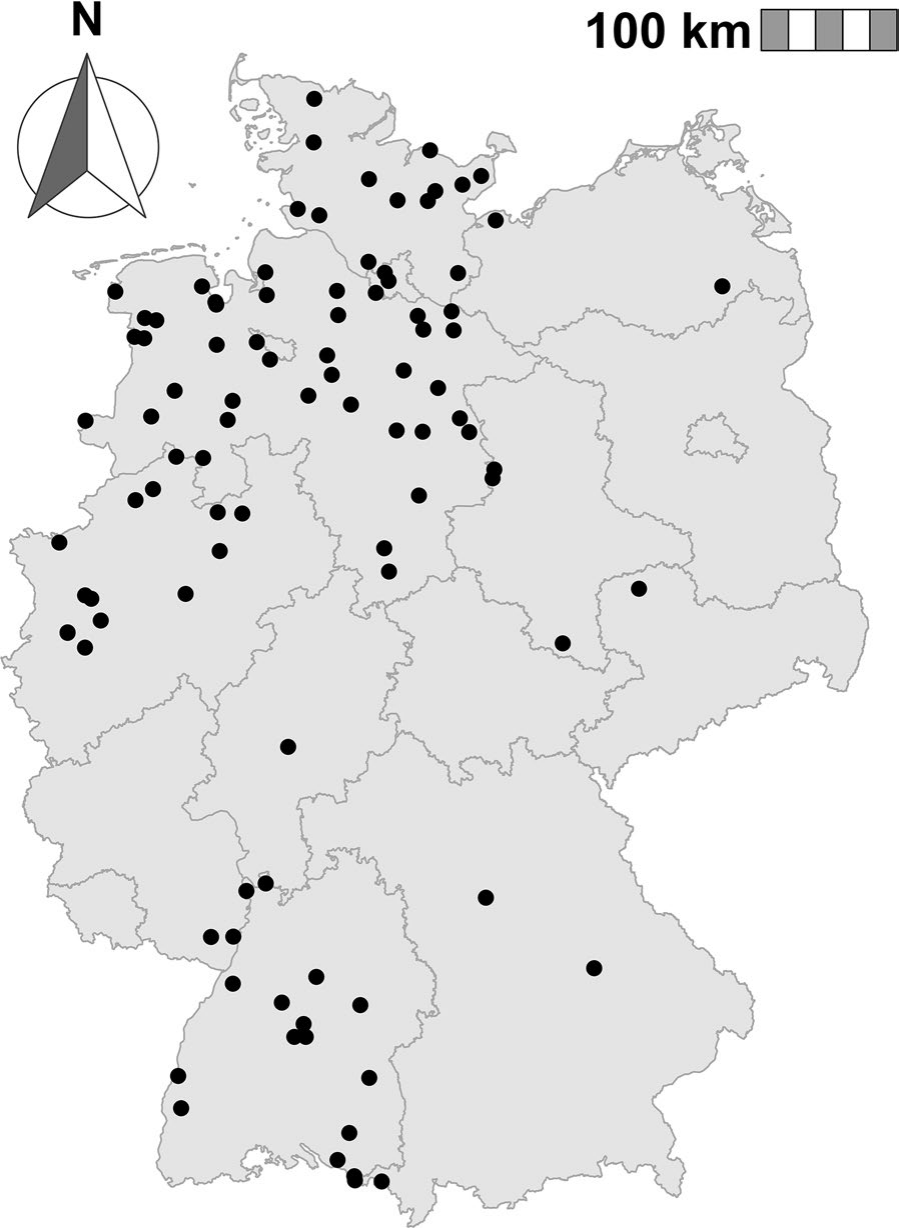
Map of Germany showing all sampling sites used to validate the process-based model

Data for the validation of the process-based model were collected with CO_2_-baited mosquito traps on a weekly, bi-weekly or daily basis from April to October in 2016–2017 and 2021–2023 (Fig. 4). In 2016, 41 sites were sampled; in subsequent years, 33 (2017), 22 (2021), eight (2022) and seven (2023) sites were sampled. All traps were run for approximately 24 h. For the 347 traps (of 3365 sampling events) that ran for > 24 h, the daily average was calculated. Five sampling sites were active across multiple years. All mosquitoes were identified by morphology using the taxonomic key by Becker et al. [1]. The blood-fed status of the caught mosquitoes was assessed using the Sella score [33], with mosquitoes categorised as unfed (Sella score 1) or as blood-fed (from freshly engorged to gravid; Sella score 2–7). Only female mosquitoes with a Sella score of 1 were used for validation, since these are the specimens considered to be host-seeking. Data from sampling sites were included in the analysis only if: the sites were sampled on at least six dates over 6 months per year, a total number of > 30 *Cx. pipiens* s.s./*Cx. torrentium* females were captured per year and > 10 mosquitoes were captured during the seasonal peak; this threshold was considered to be the minimum necessary to catch a representative seasonal dynamic. The final validation data set consisted of 106 sampling sites (Additional file 2: Tables S1, S2) with 73,540 *Cx. pipiens* s.s./*Cx. torrentium* females (mean per day = 26.69; 95% confidence interval [95% CI] 21.31−32.08). *Culex pipiens* s.s./*Cx. torrentium* females cannot be reliably distinguished by their morphology. Due to the ubiquitous distribution of *Cx. pipiens* s.s. and the fact that *Cx. torrentium* is less frequently captured using CO_2_-baited traps, the potential inclusion of *Cx. torrentium* is expected to have little effect. However, to account for this uncertainty, we refer to these specimens collectively as *Cx. pipiens* s.s./*Cx. torrentium* throughout this manuscript.

Mean daily temperature and daily rainfall data (European Reanalysis and Observations for Monitoring [EURO4M] E-OBS v29 dataset; https://www.ecmwf.int/en/forecasts/dataset/european-reanalysis-and-observations-monitoring) were obtained from the ECA&D project (http://www.ecad.eu, [34]) and extracted for each sampling site for the period 2015–2023. For each sampling site and sampling date, the sum of simulated nulliparous and parous host-seeking females was compared with data from the field-caught samples. Therefore, smoothed rolling means were calculated for the captured mosquito counts, the process-based model output and the temperature-only baseline, using a rolling window length of 4 days to reduce short-term fluctuations. For each sampling site, all smoothed time series were subsequently normalised to relative values to remove differences in absolute magnitude and to focus on temporal dynamics. Because the response variable was bounded between 0 and 1, site-level inference was based on beta regression. Two beta regression models were fitted per site, with one model relating relative observed mosquito abundance to mechanistic model output and a second model relating relative observed abundance to daily mean temperature as a baseline comparison. Temperature-only models were included to assess how well temporal patterns could be explained without an explicit population model.

A Student’s t-test was used to determine whether the correlation between the model’s simulations and actual mosquito counts from field data was significantly different from the correlation between temperature alone and field data. Validation for all available data was conducted with a beta mixed-effects model, which analysed the relative number of simulated host-seeking female mosquitoes from the model and the relative number of field-collected female mosquitoes with a Sella score of 1. Hereby, the sampling site was used as a random effect. Again, we used an additional beta mixed-effects model to examine whether the process-based model provided any advantage over simply quantifying mosquito phenology based solely on temperature data. Model performance was quantified using regression coefficients, pseudo-*R*^2^, and mean squared error (MSE) for site-specific analyses. For the pooled analysis across all sites, regression coefficients together with MSE and marginal and conditional* R*^2^ from beta mixed-effects models were used to assess model performance. A complementary scatterplot analysis of observed versus modelled relative abundance was performed at different levels of aggregation (Additional file 1: S3). Specifically, values were first analysed at the original site-specific temporal resolution, then aggregated across sites within each year by calendar week and finally aggregated across sites and years by calendar week.

### Consecutive generations per season

To account for the importance of the length of the mosquito biting season, we evaluated temperature- and photoperiod-dependent developmental transition rates within the process-based model to estimate generation turnover. For each life stage, the developmental time was calculated as the inverse of the corresponding transition rate, $\tau _i(T) = \frac{1}{\phi _i(T)}$, where $\phi _i(T)$ denotes the temperature-dependent developmental rate of each life stage. Total generation time was obtained by summing the developmental times across all life stages.

Developmental rates were evaluated using a 7-day mean temperature at the onset of each life stage. Based on the resulting total generation time, the maximum number of consecutive generations that could be completed within a season was calculated for each raster cell; these served as a proxy for the effective length of the mosquito season.

This model-based calculation was performed for each grid cell of the Europe-wide E-OBS raster for the time period 2021–2024. Due to known artefacts in the E-OBS dataset near the domain boundaries [35], maps were visually inspected and regions with implausible high or low values were excluded prior to calculating area-weighted minimum, mean and maximum numbers of consecutive generations for Europe as a whole. In addition, a buffer zone of 50 km inside national borders was applied before calculating country-level summaries for Sweden, Germany and Spain, which represent northern, central and southern European regions, respectively.

### Sensitivity analysis

We conducted a sensitivity analysis to gain insights into factors that influenced the general phenology of the population of host-seeking females (sum of nulliparous and parous females) throughout the simulation period. To this end, parameters were resampled using the Latin Hypercube Sampling, which is an efficient stratified Monte Carlo sampling method that allows for simultaneous sampling of the multidimensional parameter space [36, 37]. The simulation was carried out for 1 year, with 1000 simulations per run. Partial rank correlation coefficients (PRCCs) were computed for each selected input parameter and for the output variables (phenology of host-seeking females) for the entire simulation period.

### Software

All analysis was performed in R (version: 4.5.2) using the RStudio IDE (version: 2026.01.04) [38]. Additionally, functions from the following packages were used for data preparation, visualisation and analysis: betareg [39], DHARMa [40], performance [41], dplyr [42], tidyr [43], ggplot2 [44], scales [45], rstatix [46], stringr [47], deSolve [48], sensitivity [49], lhs [50], knitr [51], plyr [52], ggpubr [53], zoo [54], raster [55], geosphere [56], lubridate [57] and xtable [58].

## Results

### Validation

**Fig. 5:**
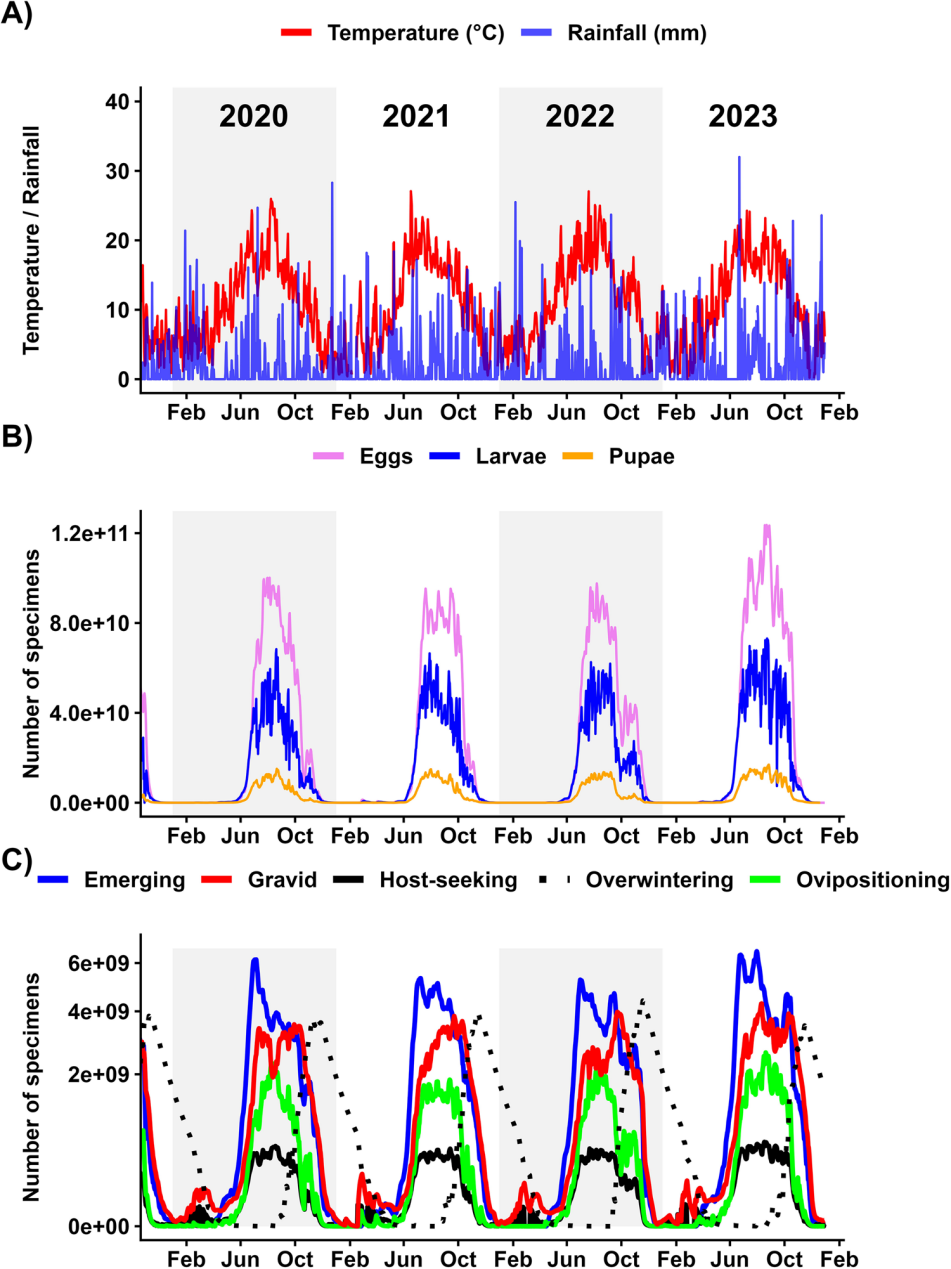
Model output for a single sampling site in Hamburg, Germany, between 2020 and 2023, with corresponding environmental data (**A**), aquatic life stages (males and females summarised) (**B**) and adult life stages (nulliparous and parous females summarised; the* Y*-axis was square-root transformed for visualisation) (**C**)

**Fig. 6:**
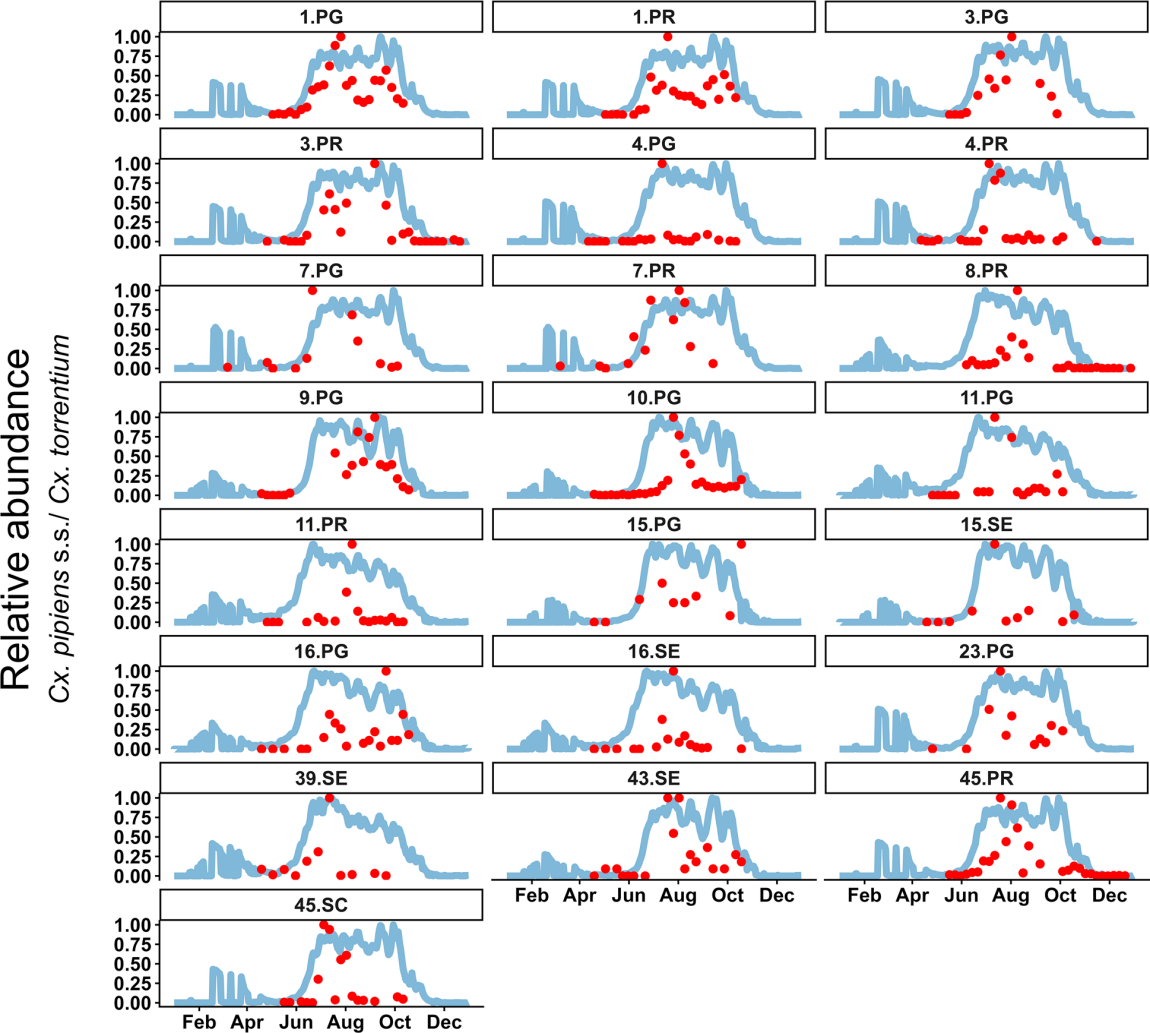
Daily model output of the relative number of host-seeking *Culex pipiens* s.s./*Culex torrentium* (blue) and relative weekly averaged field data (red) for all sampling sites in 2021. Sampling site codes indicate trap types: PG = BG-Trap Station (Gravid), PR = BG-Trap Station, SE = BG-Sentinel 2, SE = VectorCube

The process-based model depicted a seasonal dynamic for every life stage for each sampling site (Fig. 5b, c). With an average pseudo-*R*^2^-value of 0.54 (95% CI 0.49–0.59), site-specific beta regression demonstrated a significant positive association between the field data and model output for 79 sampling sites (Fig. 6; Additional file 2: Table S1); the remaining 21 sites showed no significant correlation. In contrast, site-specific beta regressions only correlating temperature with mosquito field data had an average pseudo-*R*^2^-value of 0.37 (95% CI 0.32 - 0.41). The process-based model accounts for significantly more variation in the field data than does the daily average temperature alone (*t*_()_ = 5.05, *p* < 0.0001) and showed similar model error on the relative scale ($MSE_{model}$ = 0.012; $MSE_{temperature}$ = 0.016). The beta mixed-effects model over all sites, which relates relative field captures to relative model predictions was significant (estimate [E] = 2.17, standard error [SE] = 0.062, *Z* = 35.03, *p* < 0.0001) and explained 40.42% of the variation for the fixed effect only (marginal [marg.]* R*^2^ = 0.4) and 262.06% accounting for random variation among sampling sites (conditional [cond.]* R*^2^ = 0.62). Again, the association between temperature and the field data was significant (E = 2.14, SE = 0.07, *Z* = 29.55, *p* < 0.0001), but explained less variation: 32.41% for the fixed effect (marg.* R*^2^ = 0.32) and 56.71% accounting for random variation among sampling sites (cond.* R*^2^ = 0.57). The process-based model showed a similar model error on the relative scale compared to the temperature ($MSE_{model}$ = 0.02; $MSE_{temperature}$ = 0.02).

### Consecutive generations per season

**Fig. 7:**
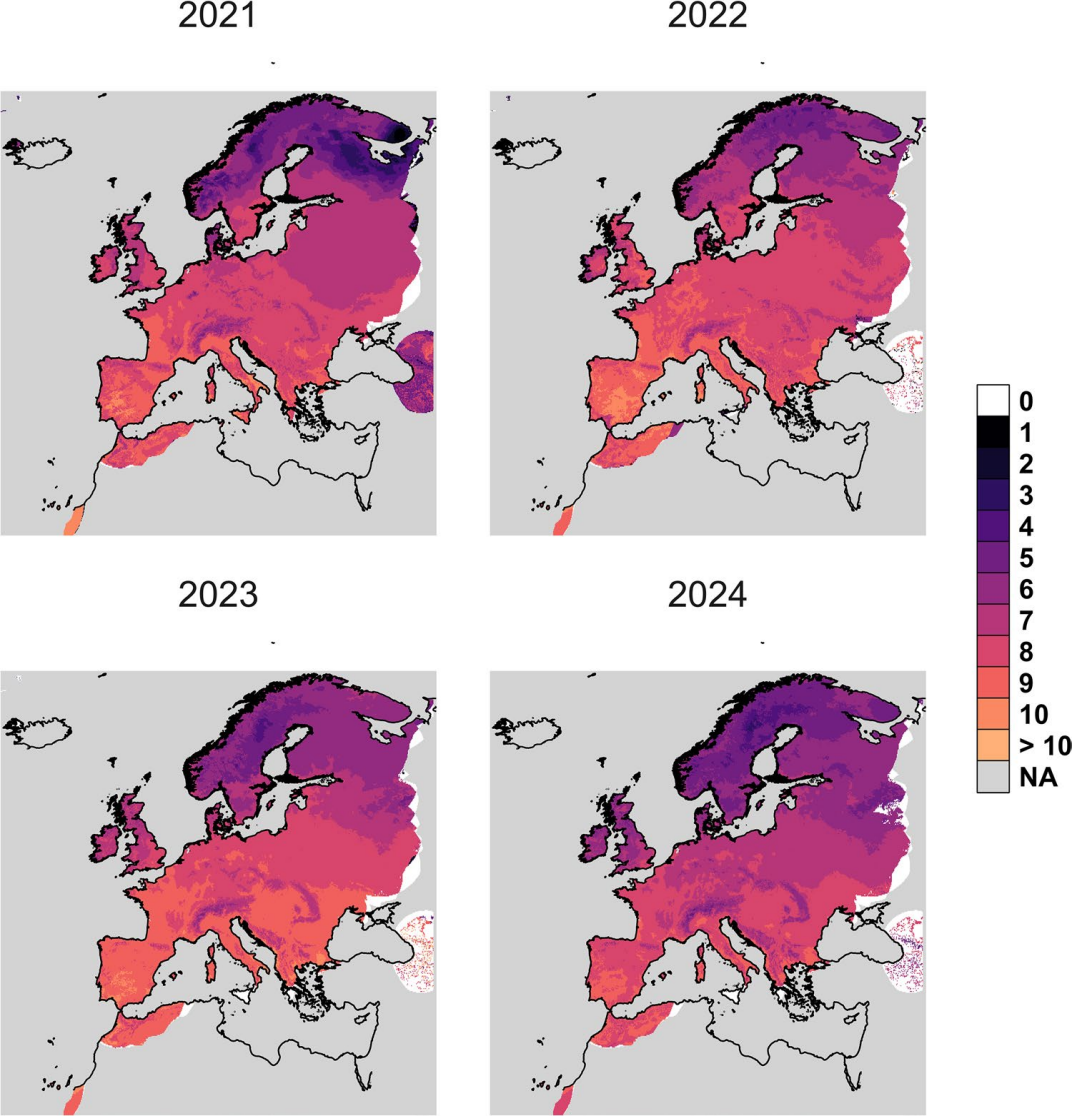
Raster map of maximum number of consecutive generations per year for *Cx. pipiens* s.s./*Cx. torrentium* over Europe

Calculation of the theoretical maximum number of consecutive generations per year for *Cx. pipiens* s.s./*Cx. torrentium* resulted in a range of one to 10 generations per year across Europe, with an average of seven generations (Fig. 7). For example, in Spain, six to 10 generations were possible, with an average of nine generations; in Sweden, three to eight generations per year were calculated, with an average of six generations; and in Germany, the number of generations ranged from five to nine, with an average of eight generations.

### Sensitivity analysis

**Fig. 8:**
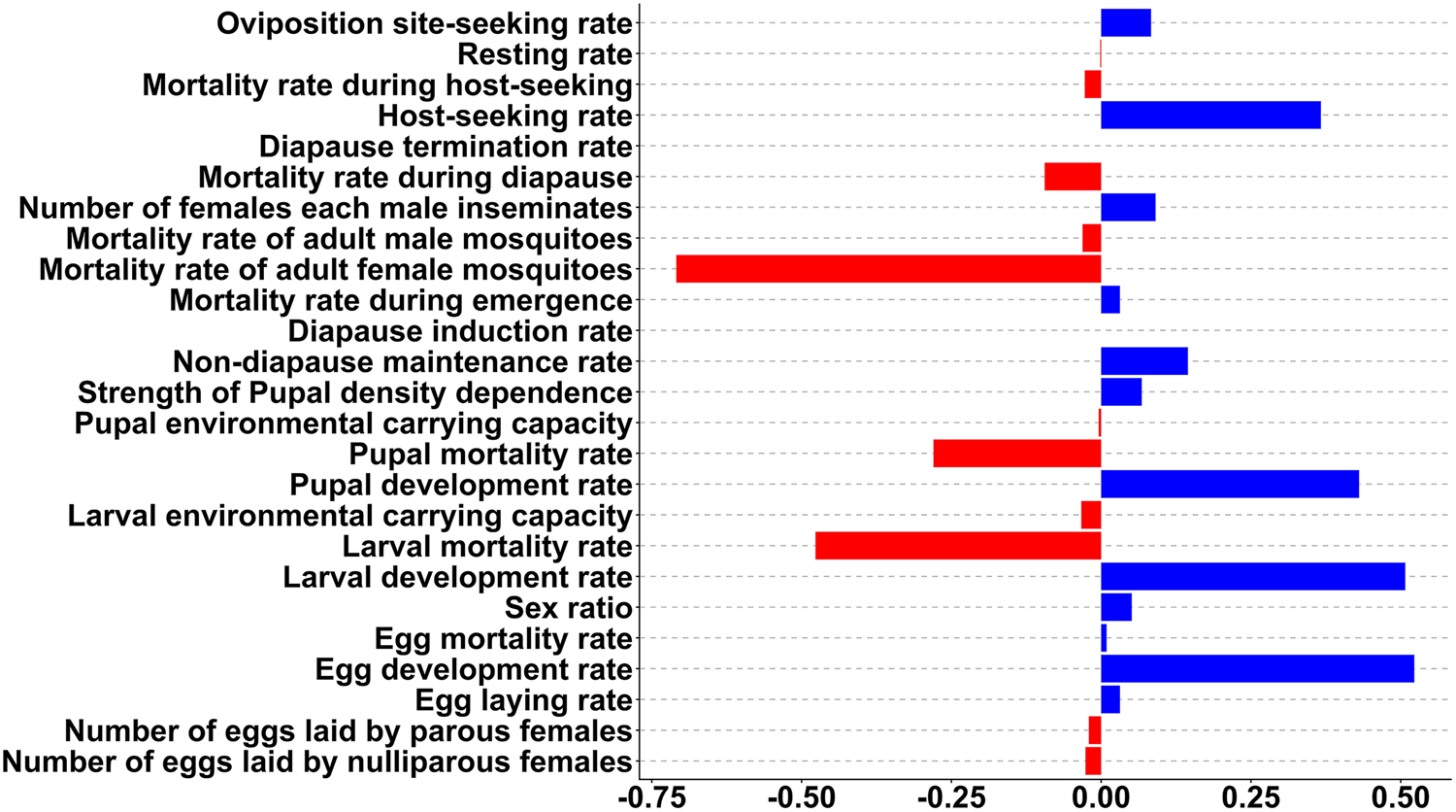
Sensitivity analysis for the phenology of host-seeking females

In the sensitivity analysis, we observed that, in particular, the egg, larval and pupal development rates as well as the host-seeking, diapause entering rate and the number of females each male inseminates are positively correlated to the phenology of the population of host-seeking adults (PRCCs > 0.2; Fig. 8). A strong negative impact (PRCCs < − 0.2) on the phenology was found for the larval and pupal mortality rate and the mortality of adult females. The egg development rate contributed the most to the phenology (PRCCs = 0.52), followed by the larval development rate (PRCCs = 0.51). The strongest negative impact on the phenology was shown for the mortality rate of adult female mosquitoes (PRCCs = − 0.71).

## Discussion

Our updated model with adapted temperature-dependent mortality and transition rates and overwintering compartment was able to depict the seasonal dynamics of *Cx. pipiens* s.s./*Cx. torrentium* for a temperate region. Although we observed strong site-specific differences in mosquito phenology, the developed process-based model was able to capture a large proportion of the observed variability. The newly added compartments, designed to integrate the overwintering period of the mosquitoes, successfully captured the mosquitoes’ behaviours observed in the field. Female *Cx. pipiens* s.s./*Cx. torrentium* overwinter, while the males do not survive [13, 30]. The inseminated females overwinter in burrows, barns and cellars and are the ones that start the new population by laying eggs in the beginning of the favourable season, after successfully feeding on a host. Photoperiod is considered to be the main trigger for the induction of the diapause [19, 20]. However, only during low temperatures was the threshold of < 15 h daylight found to induce diapause under laboratory conditions. Previous publications either relied on predefined favourable and unfavourable seasons to simulate overwintering behaviour [16] or, when diapause was explicitly modeled, it was triggered during a nulliparous stage rather than the larval stage [59]. To the authors’ knowledge, this study represents the first process-based modelling approach that explicitly captures biologically realistic overwintering dynamics in *Culex* mosquitoes. However, characterisation of the overwintering ecology of *Culex* mosquitoes still contains important knowledge gaps, particularly regarding the termination of diapause at the end of the unfavourable season; in this context, more research is needed to fully grasp the relationship between temperature and photoperiod, and the termination of diapause [21, 60]. The inclusion of the overwintering stage allowed for a dynamic start and end of the season. Since natural photoperiod variations remain constant from year to year, only temperature thresholds can postpone the induction or termination of diapause, thereby shortening or extending favourable and unfavourable seasons. The temperature-dependent transition rates were designed to produce biologically meaningful values across the entire viable temperature range, with sigmoid functions reaching an upper plateau around the temperature optima. Previously used transition rates only delivered meaningful rates in a temperature range suitable to the respective study area. On this basis, the calculated maximum number of consecutive mosquito generations has the advantage that it does not only consider the beginning and ending of the favourable season but also incorporates meteorological data of the entire season. Previous studies have also highlighted the importance of the seasonal length in disease transmission risk [61]: a longer biting season is associated with a higher transmission risk. Also, the vertical transmission of a pathogen by an infected mosquito to its offspring plays a role in the persistence of WNV in temperate regions [62]. Therefore, this process-based model and the extracted maximum number of consecutive generations provides a framework for a more realistic assessment of the local virus transmission risk.

Although not all sampling sites showed a significant association in the site-specific beta regression models, the beta mixed-effects model across all sampling sites demonstrated a significant association between relative simulated and observed host-seeking female mosquito abundance. At the same time, mosquito phenology was shown to be strongly temperature-dependent, although temperature alone explained less variation in the data set than the process-based model. The MSE differed only marginally between the process-based model and the temperature-only baseline, reflecting the strong temperature dependence of mosquito phenology, which largely determines the timing of seasonal dynamics. In contrast, the higher explained variance of the process-based model indicates that incorporating mechanistic life-cycle processes improves the representation of population structure and variability beyond temperature alone. The absence of a significant association at some sites likely reflects local heterogeneity in habitat structure, sampling intensity and microclimatic conditions that are not explicitly resolved by gridded environmental inputs, rather than a systematic failure of the model framework.

It should be noted that mosquito data collected from the field can be easily biased by various factors, such as sampling rhythms, weather conditions, trap position or errors by the volunteers running the trap, possibly leading to additional challenges in the validation of process-based models. Previous publications addressed such issues by averaging the mosquito collection data over several traps sampled on a weekly or biweekly basis and then using these data for validation on a regional scale [16–18]. In our study, we validated the model based on a large-scale data set from 106 sampling sites across Germany. We did not average the data from different sampling sites, thereby preserving the full variability of the mosquito collection data, allowing a detailed assessment of the model accuracy. Consistent with this, the scatterplot analysis (Additional file 1: Figure S3) shows that agreement between observed and modelled abundance increases with increasing levels of aggregation across sites, suggesting that discrepancies at fine temporal and spatial resolution are primarily driven by variability in the observational data rather than by structural limitations of the model. While stronger aggregation would likely improve apparent model performance, it would also mask site-specific variability and short-term dynamics. In addition, part of the mismatch at daily resolution likely arises from intrinsic oscillations of the model around the environmental carrying capacity that emerge from density-dependent processes. The remaining discrepancies therefore reflect a combination of model-inherent dynamics and environmental and sampling-related noise, including weather conditions (e.g. wind, rainfall) and variation in trap performance. Incorporating such processes explicitly, for example through an observation model or by accounting for trap success and environmental constraints, represents an important direction for future model development.

A process-based mosquito population model can be a very useful tool as it can provide valuable ecological insights, e.g. seasonal dynamics of the population or the potential maximum number of consecutive generations per season. Several parameters need further investigation to improve the accuracy of our process-based model. On the one hand, factors such as host-seeking rate or environmental carrying capacities rely on expert estimation, in contrast to temperature-dependent transition rates and mortality rates during the aquatic life stages, which could be at least experimentally measured in climate chambers. The sensitivity analysis indicates that mosquito phenology in the model is primarily driven by temperature-dependent development and mortality processes, while parameters related to host-seeking behaviour and density dependence introduce additional variability, highlighting both the biological realism of the model and key targets for future empirical refinement. Future work could explore Bayesian calibration approaches for selected parameters, potentially using reduced-form or surrogate versions of the model, to formally quantify parameter uncertainty while retaining the mechanistic structure [63]. Additional compartments for process-based mosquito population models could be considered, i.e. life stages such as resting adult male mosquitoes, however, we still lack the necessary ecological knowledge.

While precipitation was represented in the model through its effect on aquatic habitat availability, heavy rainfall events can also negatively affect immature mosquito stages through the flushing or washout of breeding sites [64]. The magnitude of such effects depend strongly on the characteristics of the breeding site (e.g. size of container habitats) and the intensity of short-term rainfall, which cannot be captured by daily mean gridded precipitation data. Incorporating rainfall-induced flushing effects therefore represents an important direction for future model development, particularly in the context of increasing rainfall variability and extreme precipitation events under climate change scenarios.

In addition, the model does not differentiate between *Cx. pipiens* s.s. and *Cx. torrentium*, two closely related sibling species which probably have different temperature dependencies [65]. Knowledge of the thermal biology of *Cx. torrentium*, in particular, is scarce, and it is not yet possible to separate these two species in our model approach. In Central and Northern Europe, these species occur sympatrically and females can only be reliably distinguished using molecular methods, as morphological characteristics alone are insufficient for female identification [1], which limits the availability of species-specific field data for model validation. Although not stated as such, previous modelling approaches for “*Culex pipiens*” in Europe treated both species collectively as *Cx. pipiens* s.s./*Cx. torrentium* because field collections were based on CO_2_-baited traps targeting females that can not be distinguished by morphological means [16, 18]. Given the predominance of *Cx. pipiens* s.s. in most regions, the potential inclusion of *Cx. torrentium* is expected to have little influence on model outcomes. This expectation is also supported by the fact that *Cx. torrentium* is disproportionately less captured by CO_2_-baited traps compared to *Cx. pipiens* s.s. [12]. Nonetheless, this simplification introduces uncertainty, especially in regions such as Northern Europe where *Cx. torrentium* occurs in high abundance [66]. Future versions of this model could benefit from integrating species-specific thermal and developmental parameters once sufficient experimental and field data become available. Such data would enable a more accurate assessment of the species’ respective ecological niches, overwintering behaviour and vector competence. Currently, however, the scarcity of field data on *Cx. torrentium* remains a major constraint for parameter differentiation and model validation..

## Conclusions

Overall, this work shows that process-based models grounded in mechanistic thermal biology, and explicitly driven by temperature, photoperiod and rainfall, can meaningfully approximate seasonal *Cx. pipiens* s.s. and *Cx. torrentium* dynamics in temperate regions, and therefore provide a useful basis for more realistic assessments of local arbovirus transmission risk.

## Supplementary Information

Additional file 1.

Additional file 2.

Additional file 3.

## Data Availability

Validation data and a minimal reproducible example are available under https://github.com/Silence1490/A-process-based-model-simulating-the-life-cycle-of-Culex-pipiens-s.s.-Cx. -torrentium-in-Germany
