# Supplementary material for: A process-based model simulating the life-cycle of Culex pipiens s.s./Cx. torrentium (Diptera: Culicidae) in Germany
Source: Parasit Vectors. 2026 May 11;19:207. doi: 10.1186/s13071-026-07410-4 (PMC13162510; doi:10.1186/s13071-026-07410-4)
Supplement: Supplementary file 1 — Additional file 1. [file 13071_2026_7410_MOESM1_ESM.pdf]

Supplementary Information

In a preliminary laboratory study, egg rafts of *Cx. pipiens* s.s./ *Cx. torrentium* were collected in Hamburg, Germany, from April to August in 2022 and 2023 and allowed to hatch under room-temperature conditions. Larvae were genetically identified using the PCR protocol described by Rudolf et al. 2013. Groups of 25 larvae were maintained in climate chambers under fluctuating temperature regimes centred at 15, 18, 21, and 24°C (± 5°C) at 70% relative humidity. Mortality and developmental time were recorded throughout the experiment to assess survival and development under temperature conditions representative of Central Europe.

Mortality varied across temperature treatments, with elevated mortality observed at lower temperatures and more variable responses at intermediate and higher temperatures (Supplementary Figure S1). These observations primarily informed the slope of the temperature-dependent mortality response and ensured biological plausibility across the implemented temperature range.

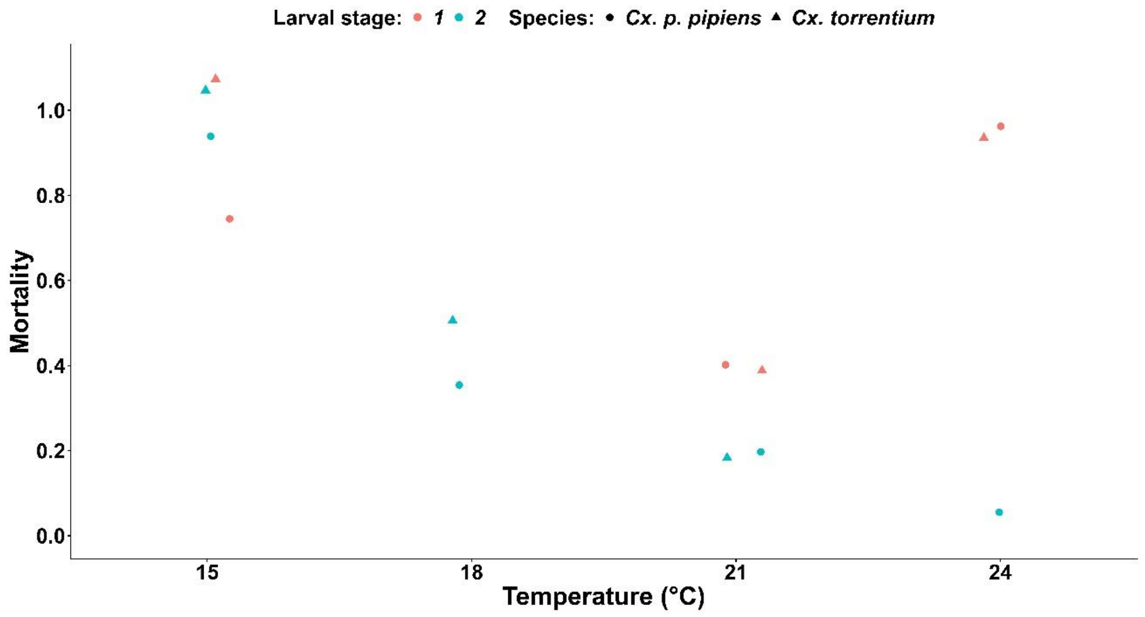

Supplementary Figure S1: Mortality of *Culex pipiens* biotype *pipiens* and *Cx. torrentium* first- and second-instar larvae under fluctuating temperature regimes (15, 18, 21, and 24°C ± 5°C) at 70% relative humidity. Each point represents the proportion of dead specimens per treatment, species and instar (n = 125 larvae per group).

Developmental time decreased with increasing temperature, with prolonged development observed at lower temperatures and more consistent, shorter development times at intermediate and higher temperatures (Supplementary Figure S2). These observations primarily informed the slope of the temperature-dependent development rates and ensured biological plausibility across the implemented temperature range.

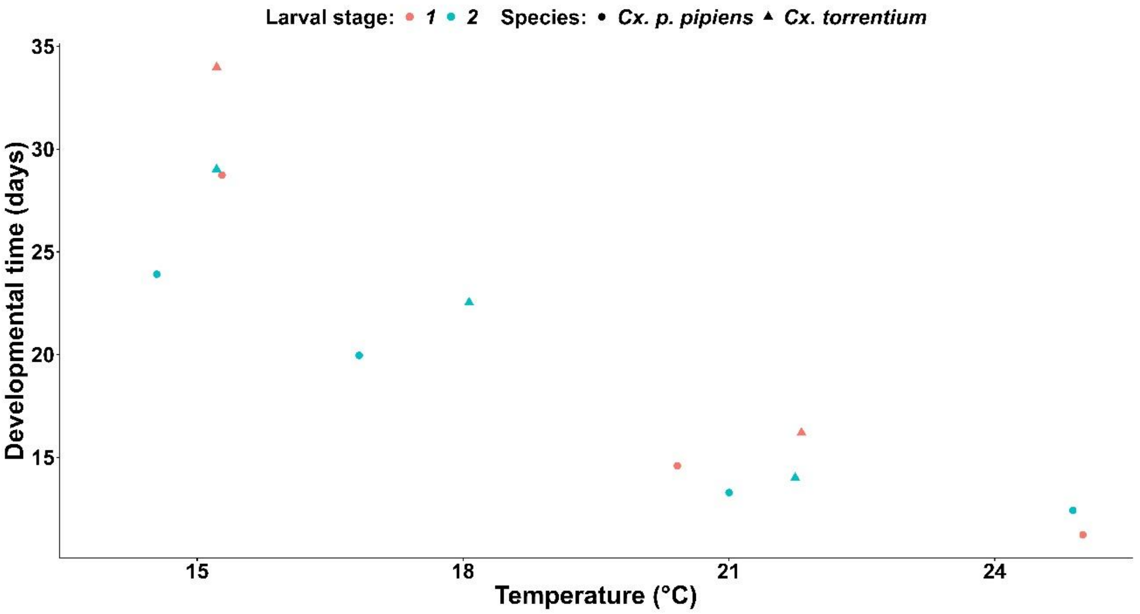

Supplementary Figure S2: Developmental time (days) of *Culex pipiens* biotype *pipiens* and *Cx. torrentium* from first- and second-instar larvae to the imago stage under fluctuating temperature regimes (15, 18, 21, and 24°C ± 5°C) at 70% relative humidity. Each point represents the mean developmental time per treatment, species, and instar.

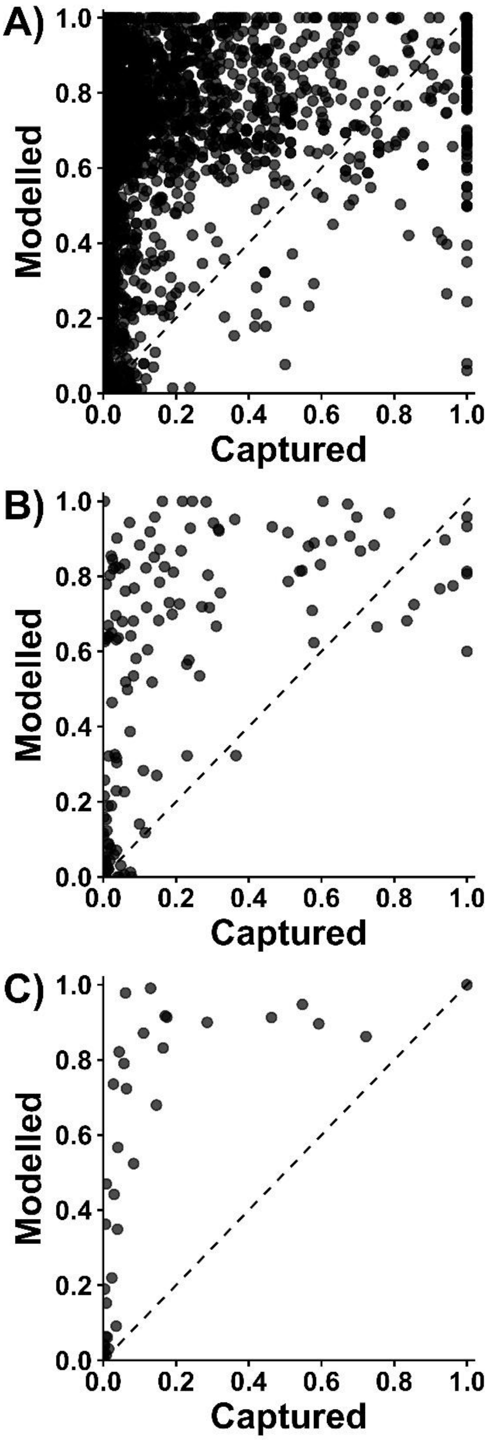

Supplementary Figure S3: *Relationship between observed (captured) and modelled relative mosquito abundance at increasing levels of aggregation. (A) Site-specific values at the original temporal resolution, (B) aggregation across sites within each year by calendar week, and (C) aggregation across sites and years by calendar week. Points represent paired observed and simulated relative abundance values. The dashed line indicates the 1:1 relationship between observations and model outputs.*

References:

Rudolf, M., Czajka, C., Börstler, J., Melaun, C., Jöst, H., von Thien, H., Badusche, M., Becker, N., Schmidt-Chanasit, J., Krüger, A., Tannich, E., & Becker, S. (2013). First Nationwide Surveillance of Culex pipiens Complex and Culex torrentium Mosquitoes Demonstrated the Presence of Culex pipiens Biotype pipiens/molestus Hybrids in Germany. PLoS ONE, 8(9), e71832. https://doi.org/10.1371/journal.pone.0071832
